# Characterization of mannanase from *Bacillus circulans* NT 6.7 and its application in mannooligosaccharides preparation as prebiotic

**DOI:** 10.1186/s40064-015-1565-7

**Published:** 2015-12-14

**Authors:** Phanwipa Pangsri, Yotthachai Piwpankaew, Arunee Ingkakul, Sunee Nitisinprasert, Suttipun Keawsompong

**Affiliations:** Department of Biotechnology, Faculty of Agro-Industry, Kasetsart University, Bangkok, Thailand; Interdisciplinary Program in Genetic Engineering, Graduate School, Kasetsart University, Bangkok, Thailand; Department of Biochemistry, Faculty of Science, Kasetsart University, Bangkok, Thailand; Special Research Unit: Probiotic and Prebiotics for Health, Center for Advanced Studies for Agriculture and Food (CASAF), Institute for Advanced Studies, Kasetsart University, Bangkok, Thailand; Center for Agricultural Biotechnology (CAB), Kasetsart University, Kamphaeng Saen Campus, Kamphaeng Saen, Nakhon Pathom, Thailand

**Keywords:** Mannanase, Defatted copra meal, Prebiotics, Mannooligosaccharides, *Bacillus circulans*

## Abstract

This study focused on the characterization of mannanase from *Bacillus circulans* NT 6.7 for mannooligosaccharides (MOS) production. The enzyme from *B. circulans* NT 6.7 was produced using defatted copra meal as a carbon source. The mannanase was purified by ultrafiltration and column chromatography of Q-Sepharose. The purified protein (M1) was a dimeric protein with a 40 kDa subunit. The purified M1 exhibited optimum pH and temperature at pH 6.0 and 60 °C, respectively. It was activated by Mn^2+,^ Mg^2+,^ and Cu^2+^, and as inhibited by EDTA (45–65 %). The purified enzyme exhibited high specificity to beta-mannan: konjac (glucomannan), locust bean gum (galactomannan), ivory nut (mannan), guar gum (galactomannan) and defatted copra meal (galactomannan). The defatted copra meal could be hydrolyzed by purified M1 into mannooligosaccharides which promoted beneficial bacteria, especially Lactobacillus group, and inhibited pathogenic bacteria; *Shigella dysenteria* DMST 1511, *Staphylococcus aureus* TISTR 029, and *Salmonella enterica serovar Enteritidis* DMST 17368. Therefore, the mannanase from *B. circulans* NT 6.7 would be a novel source of enzymes for the mannooligosaccharides production as prebiotics.

## Background

In 2004, “prebiotic” was defined as ‘‘selectively fermented ingredients that allow specific changes, both in the composition and/or activity in the gastrointestinal microbiota that confers benefits upon host well-being and health” (Gibson et al. 2004). Bifidobacteria and Lactobacilli are probiotic microbials that benefit the host by improving intestinal microbial balance (Gibson and Roberfroid 1995). Pathogenic bacteria (*Clostridiabacteroides*, *Escherichia coli* and *Salmonella*) produce toxins which can affect human gastrointestinal tracts, resulting in diarrhea, vomiting and nausea (Manning 2004). Some prebiotics, such as inulin, galactooligosaccharides (GOS), fructooligosaccharides (FOS), xylooligosaccharides (XOS), and mannooligosaccharides (MOS), are widely used in the market.

Mannooligosaccharides are derived from the cell wall of the yeast *Saccharomyces cerevisiae* is commercially available as a feed supplement (Ferket et al. 2002). However, they can also be produced from plant mannan, such as konjac, ivory nut, locust bean, palm kernel, coffee bean, and copra meal. Copra meal is a by-product of the coconut milk process and contains a large amount of mannose in the form of mannan. Mannan consists of repeating β-1, 4 mannose units and a few α-1, 6-galactose units attached to a β-1, 4 mannose backbone (Mohammad et al. 1996).

Mannanase was classified, based on the site of lysis in hydrolytic process, into two types: endo-β-1, 4-mannanase EC 3.2.1.78) and β-mannosidase (EC 3.2.1.25). Endo-β-1, 4-mannanase can randomly cleave bonds within mannan chain while β-mannosidase enzyme is capable of removing one or more mannose units from the ends of chains. Mannanase can hydrolyze copra meal into mannooligosaccharides. The effects of copra mannan hydrolysates in gastrointestinal tracts are to stimulate the growth of intestinal microflora and limit pathogenic bacteria. (Titapoka et al. 2008). Mannanases are produced by various microorganisms, including bacteria, yeast, and fungi. In particular, mannanase from *Bacillus circulans* NT 6.7 can hydrolyze mannan into mannooligosaccharides. Hydrolysates can inhibit the growth of pathogens (*Salmonella serovarEnteritidis* S003 and *E. coli* E010) and can promote the growth of probiotic bacteria (*Lactobacillusreuteri* KUB-AC5) (Phothichitto et al. 2006). This result suggests that mannanase from *B. circulans* NT 6.7 is suitable for the preparation of mannooligosaccharides. Therefore, this research aimed to characterize the purified mannanase from *B. circulans* NT 6.7 and its application in the preparation of mannooligosaccharides as prebiotics.

## Results and discussion

### Production and purification of mannanase

Defatted copra meal was used for mannannase production in a producing medium in 5–l fermenter cultivation: 600 rpm, 0.75 vvm and 45 °C. *B. circulans* NT 6.7 exhibited the highest cell growth and mannanase activity with 2.43 × 10^9^ CFU/mL and 27.70 units/mL respectively at 6 h. The crude enzyme was concentrated 10× by ultrafiltration and applied to anion-exchange chromatography. The result showed that the purified mannanase from Q-Sepharose chromatography had 628-folds of purification and specific activity of 295 units/mg protein (Table 1). The purified protein was named M1.

**Table 1:** Purification table of mannanase from *B. circulans* NT 6.7

Step	Total volumn (mL)	Total enzyme activity (unit)	Total protein (mg)	Specific activity (unit/mg protein)	Purifi cation (fold)	Yield (%)
Crude enzyme	1000	22,640	47,900	0.47	1.00	100
UF	100	4185	3985	1.05	2.23	18.48
Anion exchange (Q-Sepharose)	45	61.92	0.21	295.0	628.0	0.27

**Fig. 1:**
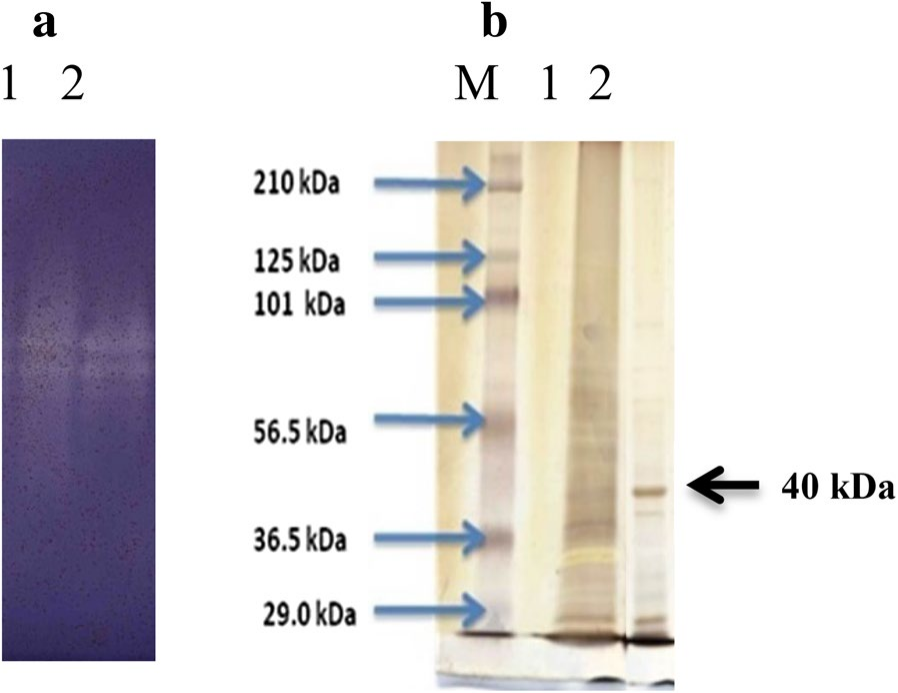
Zymogram analysis and SDS-PAGE of purified M1 **a** zymogram analysis: *lane* 1 crude enzyme *lane* 2, purified M1; **b** SDS-PAGE: *lane* M Marker (Bio-Rad, USA) *lane* 1 crude enzyme *lane* 2 purified M1

The molecular weight of purified M1 were 39.80 and 75.85 kDa, by gel filtration and the zymogram of purified M1 also showed two bands in active gel (Fig. 1a). N-terminal sequences of these two fractions (39.80 and 75.85 kDa) were the same and the SDS-PAGE showed the single band at 40 kDa as shown in Fig. 1b. Therefore, the purified M1 could be a dimeric protein containing two subunits (40 kDa). The result was consisted with Piwpankaew et al. (2014) reported that the mannanase gene of *B. circulans* NT 6.7 consisted of 1083 nucleotides encoding 360 amino acid residues with theoretical molecular weight 40.29 kDa. The purified M1 also had the same molecular weight as other *Bacillus* species; Zakaria et al. (1998) reported that the mannanase from *Bacillus subtilis* KU-1 had a molecular weight of 39 kDa. The purified MAN 5 had a single band on SDS-PAGE at 40.5 kDa (Zhang et al. 2009).

### Characterization of the purified mannanase

**Fig. 2:**
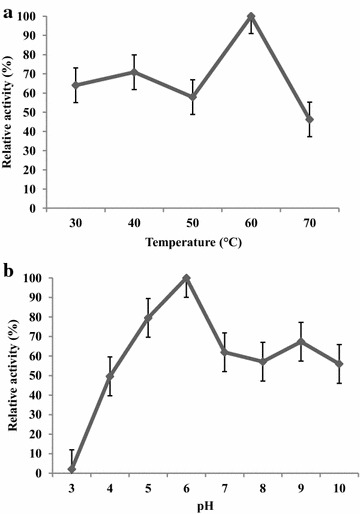
Effect of temperature (**a**) and pH (**b**) on purified M1

The effects of temperature on enzyme activity were determined for the purified M1. The optimum temperature was measured using the standard assay while varying the temperature from 30 to 70 °C. The optimum temperature was 60 °C for the 60 min assay (Fig. 2a). The effects of the pH value on β-mannanase activity were determined for purified enzyme using the standard assay and varying the pH from 3.0 to 10.0. The optimum pH β-mannanase activity was 6.0 (Fig. 2b).

These results were similar to the results of Phothichitto et al. (2006), who reported the optimum pH and temperature of the crude enzyme from *B. circulans* NT 6.7 were pH 6.0 and 50 °C, respectively. Piwpankaew et al. (2014) also reported the optimum pH and temperature of recombinant β-mannanase of *B. circulans* NT 6.7 were pH 6.0 and 50 °C, respectively. The optimum pH and temperature of the purified M1 were similar to those of other bacterial mannanases. Mohammad et al. (1996) showed the optimum temperature of purified mannanase from *Bacillus* sp. KK01 was 60 °C. The purified mannanase from *Bacillus licheniformis* had optimum temperature and pH at 60 °C and pH 7.0 (Zhang et al. 2000). Therefore, the mannanase was optimum temperature at high temperature, making the enzyme more attractive for industrial applications.

The purified M1 was tested for substrate specificity; locust bean gum, alpha-mannan, ivory nut mannan, konjac mannan, guar gum, oat spelts xylan, birchwood xylan, carboxymethylcellulose (CMC), avicel, copra meal and defatted copra meal. The purified M1 exhibited high specificity for konjac mannan (glucomannan), locust bean gum (galactomannan), ivory nut (mannan), guar gum (galactomannan) and defatted copra meal (galactomannan). These results indicate that the purified M1 was specific and exhibited high activity on β-mannan substrates as shown in Table 2. The result was similar with mannanase from *B. subtilis* KU1, which specifically degraded only konjac mannan, locust bean gum and guar gum (Zakaria et al. 1998).

**Table 2:** Substrate specificity of purified M1

Substrate	Relative activity (%)
Konjac mannan	100
Locust bean gum	50.04
Ivory nut	17.56
Guar gum	5.62
Defatted copra meal	4.81
Xylan from birchwood	2.39
CMC	1.97
Xylan from oat spelt	1.51
Alpha mannan	1.41
Avicel	<1

The effect of various metal ions and EDTA on the purified M1 activity was tested by adding 1 mM of the selected metal ions or EDTA to the enzyme reactions. The mannanase activity (M1) was activated by Mn^2+^ (123.53 %), Cu^2+^ (121.53 %) and Mg^2+^ (116.26 %). While, the purified M1 was inhibited 45–65 % by EDTA, which indicated that the purified M1 could be a metalloenzyme (Table 3). The magnesium ion is the most widespread metal present in enzymes. Manganese ions function as enzyme activators and components of metalloenzymes (enzymes that contain a metal ion in their structure), and Cu^2+^ is a metal ion that functions well as a redox center. Magnesium plays a central role as the essential partner of phosphate-containing substrates, including ATP, which is largely present in cells as Mg^2+^ complex (Luthi et al. 1999).

**Table 3:** Effect of metal ions and EDTA on purified M1 activity

Metal ions	Relative activity of M1 (%)
Control	100
CoCl_2_	100.02
CaCl_2_	95.80
FeSO_4_	97.75
MnSO_4_	123.53
ZnSO_4_	105.04
MgSO_4_	100.02
CuSO_4_	121.53
LiCl_2_	102.93
MgCl_2_	116.26
EDTA	61.35
Urea	94.32
β-Mercaptonethanol	107.13
SDS	85.07

### Hydrolysis Product of mannan

The purified M1 could hydrolyze locust bean gum (LBG) into mannobiose, mannotriose, mannotetraose, mannopentaose, and mannohexaose (M2–M6), while konjac mannan and defatted copra meal were hydrolyzed into mannotriose, mannotetraose mannopentaose and mannohexaose (M3–M6) (Table 4). The purified M1 catalyzed the random cleavage of β-1, 4-mannosidic linkages in the mannan backbone into mannooligosaccharides of various sizes. In addition, the purified M1 had the ability to hydrolyze randomly within the 1, 4-β-d mannan main chains of different mannan sources with different mannan backbones. Locust bean gum had a mannose: galactose ratio of 4:1, konjac mannan composed of mannose and glucose (3:1) and copra meal had linear β-1, 4-mannan backbones with only a few β-galactosyl substituted. The result, as shown in Table 4, showed that defatted copra meal hydrolysate had a higher amount of mannooligosaccharides than konjac mannan and locust bean gum hydrolysates, respectively. The purified M1 hydrolyzed defatted copra meal, which had a linear β-1, 4-mannan backbone with only a few β-galactosyl stubstituted, into mannooligosaccharides; mainly manotriose, mannotetraose and mannopentaose. These results correlated with those of Titapoka et al. (2008), who reported that the products hydrolyzed from copra meal by the S1 enzyme were mannotriose and mannotetraose. As copra meal was incubated with the *B. subtilis* WY34 mannanase, mannnotetraose, mannotriose, and mannobiose were produced (Jiang et al. 2006).

**Table 4:** Hydrolysis product of mannan hydrolysed by the purified M1

Substrate	Products (mg/mL)					
	Manno hexaose	Manno pentaose	Manno tetraose	Manno triose	Manno biose	Mannose
Konjac	0.62	0.49	0.34	0.12	–	–
Locust bean gum	2.45	0.8	0.34	0.18	0.06	–
Defatted copra meal	0.85	1.58	1.45	1.98	–	–

1 % substrates of konjac mannan, locust bean gum and defatted copra meal were hydrolyzed by the purified M1 at pH 6.0 at 60 °C for 120 min

The purified M1 could hydrolyze konjac mannan, locust bean gum and defatted copra meal into mannooligosaccharides. However, the ratio of mannobiose, mannotriose, mannotetraose, mannopentaose, mannohexaose (M2–M6) depended on the substrates. Moreover, the purified M1 tended to hydrolyze galactomannan (locust bean gum and defatted copra meal) into mannooligosaccharides more often than glucomannan (konjac mannan). Therefore, purified M1 could be galacto-mannanase, which would be suitable for mannooligosaccharide preparation from copra meal (galactomannan).

### Enhancement/inhibition properties of defatted copra meal-hydrolysate (DCM-hydrolysate)

**Fig. 3:**
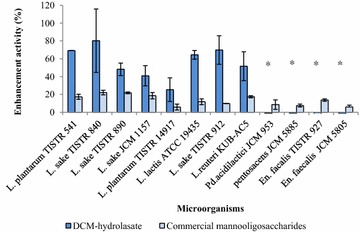
Enhancement properties of DCM-hydrolysate prepared with the purified M1 on beneficial bacteria

**Fig. 4:**
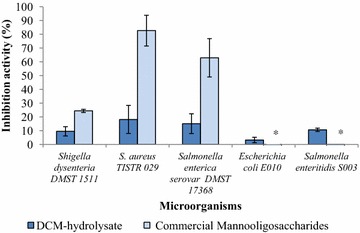
Inhibition properties of DCM-hydrolysate prepared with the purified M1 on pathogenic bacteria

The results demonstrated that DCM-hydrolysate and commercial mannooligo-saccharide from yeast cell walls could support the growth of beneficial bacteria. It was shown that probiotic bacteria could use the oligosaccharides in DCM-hydrolysate and commercial mannooligosaccharide to support their growth. DCM-hydrolysate could promote the growth of *Lactobacillus* sp. (8 strains) (Fig. 3) and inhibit the growth of pathogenic bacteria (5 strains) as shown in Fig. 4. This result was similar to the report by Phothichitto et al. (2006), in which the culture filtrate of *B. circulans* NT. 6.7 grown on copra meal could promote the growth of *L. reuteri* AC-5 up to 49.593 %, but could not inhibit *S. serovar Enteritidis* S003 or *E. coli* E010. Titapoka et al. (2008) reported that the copra meal hydrolysate by mannanase from *Klebsiella oxytoca* KUB-CW2-3 showed higher enhanced activity for the growth of *L. reuteri* KUB-AC5. The commercial mannooligosaccharides from yeast cell walls could stimulate the growth of beneficial bacteria and inhibit the growth of three strains of pathogenic bacteria (*Shigella dysenteria* DMST 1511, *Staphylococcus aureus* TISTR 029, and *Salmonella enterica serovar Enteritidis* DMST 17368). This result was similar to that of Line et al. (1997), who reported that yeast cell wall carbohydrates, especially mannose residue, were effective in preventing *Salmonella* sp. colonization. Newman (1994) reported that MOS derived from the outer cell wall of selected *S. cerevisiae* strains had the ability to adhere to pathogenic bacteria, such as *Salmonella* or *E. coli*. These results suggest that defatted copra meal-hydrolysate from purified M1 has the ability to promote beneficial bacteria and inhibit pathogenic bacteria. Therefore, defatted copra meal-hydrolysate from purified M1 could be a novel candidate for a prebiotic.

## Conclusions

Defatted copra meal can be used as a substrate for mannanase production and mannooligosaccharide preparation. The purified M1 from *B. circulans* NT 6.7 was a dimeric protein and the pH and temperature optimum were 6.0 and 60 °C, respectively. The purified M1 was specified to mannan substrates; konjac mannan, locust bean gum, ivory nut, guar gum and defatted copra meal. The M1 was activated by Mn^2+^, Mg^2+^, and Cu^2+^ and inhibited by EDTA. The purified M1 hydrolyzed konjac mannan, locust bean gum and defatted copra meal into mannooligosaccharides. The ratio of mannobiose, mannotriose, mannotetraose, mannopentaose, mannohexaose (M2–M6) was dependent on the substrates and tended to hydrolyze galactomannan (locust bean gum and defatted copra meal) into mannooligosaccharides rather than glucomannan (konjac). Therefore, purified M1 could be galacto-mannanase, which is suitable for mannooligosaccharide preparation from copra meal. The defatted copra meal hydrolysate could effectively promote the growth of beneficial bacteria and inhibit pathogenic bacteria more than commercial mannooligosaccharides, prepared from yeast cell wall. These results thus provide preliminary data regarding the potential of the defatted copra meal hydrolysate as a novel candidate for a prebiotic.

## Methods

### Microorganism and enzyme production

*Bacillus circulans* NT. 6.7 (Phothichitto et al. 2006) was cultivated in a producing medium: 1 % defatted copra meal, 3 % peptone, 1.5 %, KH_2_PO_4_, 0.06 %, MgSO_4_·7H_2_O, and 2.5 % corn steep liquor at pH 7.0 in a 5-l fermenter (Biostat-B). The cultivation was conducted at agitation speeds of 600 rpm, aeration 0.75 vvm (Feng et al. 2003) at 45 °C (Phothichitto et al. 2006) for 6 h. Cells were removed by centrifugation at 8000*g* for 15 min at 4 °C, and the supernatant was assayed for mannanase activity.

### Enzyme assay

The β-mannanase was assayed by incubating a reaction mixture of 0.5 mL of sample and 0.5 mL of 50 mM potassium phosphate buffer at pH 7.0 with 1 % (w/v) locust bean gum at 60 °C for 60 min. The amount of reducing sugar released was determined by 3, 5-dinitrosalicylic acid (DNS) method using d-mannose as the standard (Miller 1959).

One unit of enzyme activity was defined as the amount of enzyme that produced 1 μm of reducing sugar per minute under the experimental conditions.

### Protein assay

The protein concentration was determined by the method of Lowry et al. (1951). Bovine serum albumin was used as a standard.

### Purification of Mannanase

The crude enzyme was concentrated 10× by ultrafiltration using a 10 kDa Mw cut-off membrane (Minimate TFF System, PALL, USA). Two mL of concentrated enzyme were applied to 1.6 × 20 cm Q-Sepharose column (Titapoka et al. 2008) and equilibrated with 20 mM Tris–HCl buffer (buffer A) at pH 8.5, with a flow rate of 3 mL/min. The column was washed with buffer A until there was no eluting protein. Then, the protein was eluted with 0–1 M sodium chloride in 20 mM Tris–HCl buffer (buffer B) at pH 8.5, eluting protein was detected by absorbance measurements at 280 nm. The fractions were collected using a fraction collector (ÄKTA explorer, GE Healthcare Life Sciences, Sweden). All fractions were measured for mannanase activity.

### Determination of Molecular weight

The molecular weight of purified protein was determined by gel filtration (Sephacryl S-100, (GE healthcare). The maker of gel filtration calibration kit LMW (low molecular weight) use composed of aprotinin (6500 kDa), ribonuclease (13,700 kDa), carbonic anhydrase (29,000 kDa), ovalbumin (43,000), conalbumin (75,000 kDa) and Blue Dextran 2000 (GE healthcare).

The molecular weight and purity of the purified enzyme were also determined by SDS-PAGE according to Laemnli (1970) using 10 % acrylamide separating gel and stained with silver stain plus kit (Bio-Rad). The pre-stained marker (Bio-Rad) composed of myosin (195,755), β-galactosidase (107,181), bovine serum albumin (59,299), ovalbumin (41,220), carbonic anhydrase (27,578), soybean trypsin inhibitor (20,514), lysozyme (15,189) and aprotinin (6458) was used.

### Zymogram analysis

To confirm the activity of the purified proteins, native protein samples were separated on a 10 % acrylamide separating gel. Then, the native gel was incubated at 50 °C for 1–2 h. Congo red 0.1 % was used for staining. Congo red was washed by 1 M NaCl and the clear zone activity was fixed with 5 % acetic acid. Enzyme activity on the substrate gel was visualized as a clear zone against a blue background.

### Determination of optimum pH and optimum temperature

The optimum pH and temperature of enzyme activity were determined at pH 3.0, 4.0, 5.0, 6.0, 7.0, 8.0, 9.0 and 10.0 at 30, 40, 50, 60 and 70 °C, respectively. The following 50 mM buffer solutions were used: citrate (pH 3.0–6.0), phosphate (pH 6.0–8.0) and glycine-NaOH (pH 8.0–10.0).

### Determination of pH stability and temperature stability

The effect of pH on enzyme stability was determined at pH 3.0–10.0 in 50 mM buffer: citrate (pH 3.0–6.0), phosphate (pH 6.0–8.0) and glycine-NaOH (pH 8.0–10.0). The enzyme solution was incubated in the buffer system at 60 °C for 60 min. The remaining enzyme activity was measured at temperature optimum for 60 min. Thermal stability of the enzyme was determined at 30, 40, 50, 60, 70, and 80 °C in 50 mM buffer at pH 6.0. After 60 min, the remaining enzyme activity was measured.

### Effect of metal ions on enzyme activity

The effects of metal ions Li^+^, Ca^2+^, Cu^2+^, Fe^2+^, Mg^2+^, Mn^2+^, Zn^2+^, Ni^2+^, Co^2+^, urea, SDS, EDTA and β-mercaptoethanol on the enzyme activity were determined in the presence of 1 mM of each ion under optimum conditions.

### Determination of substrate specificity

The activity of the purified enzyme was determined as previously described under optimum conditions on 1.0 % (w/v) substrates: locust bean gum (Sigma), alpha-mannan (Megazyme), ivory nut mannan (Megazyme), konjac mannan (Megazyme), guar gum (Megazyme), xylan (from oat spelts) (Sigma), xylan (from birchwood) (Sigma), carboxymethylcellulose (CMC) (Fluka), avicel (Megazyme), copra meal and defatted copra meal.

### Determination of hydrolysis product

The 1 % substrates of konjac mannan, locust bean gum and defatted copra meal were hydrolyzed by the purified M1 at pH 6.0 at 60 °C for 120 min. The hydrolysis products were analyzed by HPLC under the following conditions: column, Aminex-HPX42C; mobile phase, DI water; column temperature, 75 °C; flow rate, 0.4 mL/min; and refractive index detector. The mannose (Fluka), mannobiose, mannotriose, mannotetraose, mannopentaose and mannohexaose (Megazyme) were used as standards.

### Enhancement/inhibition properties of defatted copra meal hydrolysate on beneficial bacteria/pathogenic bacteria

Defatted copra meal hydrolysate was prepared under optimal conditions three trials. 12 strains of beneficial bacteria and five strains of pathogenic bacteria were cultivated in 5 mL of media with 1 % DCM-hydrolysate or commercial mannooligosaccharides (yeast cell wall). *Lactobacillus* and *Pediococcus* were cultivated in 5 mL of MRS broth and BHI broth (Agnes et al. 2005) respectively, at 37 °C for 4 h under anaerobic conditions. *Enterococcus* and pathogenic bacteria were cultivated in 5 mL of BHI broth and NB medium, respectively, at 37 °C under aerobic conditions for 4 h. Cell growth was determined by measuring optical density at 600 nm. The enhancement and inhibition activities were calculated by the following equations (Phothichitto et al. 2006):

$${\text{Enhancement activity }}\left ( \% \right) = \frac{(SB - CB)}{CB} \times 100$$

$${\text{Inhibition activity }}\left ( \% \right) = \frac{(CB - SB)}{CB} \times 100$$

SB is the optical density of cell in medium with DCM-hydrolysate product/commercial mannooligosaccharides.

CB is the optical density of cell in medium without DCM-hydrolysate product/commercial mannooligosaccharides.
